# Combined angiography and perfusion using radial imaging and arterial spin labeling with structural contrast

**DOI:** 10.1002/mrm.70073

**Published:** 2025-09-15

**Authors:** Thomas W. Okell, Joseph G. Woods, Mark Chiew

**Affiliations:** ^1^ Oxford Centre for Integrative Neuroimaging, FMRIB, Nuffield Department of Clinical Neurosciences University of Oxford Oxford UK; ^2^ Department of Diagnostic, Interventional and Pediatric Radiology University of Bern Bern Switzerland; ^3^ Physical Sciences, Sunnybrook Research Institute Toronto Ontario Canada; ^4^ Department of Medical Biophysics University of Toronto Toronto Ontario Canada

**Keywords:** arterial spin labeling, dynamic angiography, non‐contrast MRI, perfusion imaging, simultaneous acquisition, T_1_‐weighted structural imaging

## Abstract

**Purpose:**

To develop a non‐contrast MRI method for the simultaneous acquisition of time‐resolved 3D angiographic, perfusion, and multi‐contrast T_1_‐weighted structural brain images in a single 6 min acquisition.

**Methods:**

The proposed combined angiography and perfusion using radial imaging and arterial spin labeling with structural contrast (CAPRIA+S) pulse sequence uses pseudocontinuous arterial spin labeling to label inflowing blood, an inversion pulse to provide background suppression and T_1_‐weighted contrast, and a continuous 3D golden ratio spoiled gradient echo readout. Label‐control subtraction isolates the blood signal which can be flexibly reconstructed at high/low spatiotemporal resolution for angiography/perfusion imaging. The mean signal retains the static tissue, allowing T_1_‐weighted structural images to be reconstructed at different effective TIs. CAPRIA+S was compared with conventional time‐of‐flight angiography, 3D‐gradient and spin echo pseudocontinuous arterial spin labeling perfusion imaging, and MPRAGE structural imaging (10 min total) in healthy volunteers.

**Results:**

CAPRIA+S gave improved distal vessel visibility and fewer artifacts than time‐of‐flight angiography, while also providing dynamic information, with blood transit time and dispersion maps. CAPRIA+S perfusion images were comparable to 3D‐gradient and spin echo data but without through‐slice blurring or artifacts in inferior brain regions. Comparable quantitative cerebral blood flow maps were produced, with CAPRIA+S being significantly more repeatable. Structural CAPRIA+S images were comparable to MPRAGE but also yielded a range of T_1_‐weighted contrasts and allowed quantitative T_1_ maps to be estimated.

**Conclusion:**

CAPRIA+S is an efficient single acquisition to provide intrinsically co‐registered quantitative information about brain blood flow and structure that has considerable advantages over conventional methods.

## 1 INTRODUCTION

The use of angiography to visualize blood flow through the brain‐feeding arteries is vital in many cerebrovascular diseases, allowing the assessment of stenoses, occlusions, or abnormal vessels such as arteriovenous malformations. However, the effect of any blood flow disruptions on downstream tissue perfusion is also important to observe because collateral flow and other homeostatic mechanisms can compensate for compromised arterial flow. In many clinical and research settings, structural images are also required to assess perfusion abnormalities relative to brain structure, to look for abnormal tissues and co‐register data from multiple subjects.

Arterial spin labeling (ASL)^1^, ^2^ is an MRI‐based approach capable of generating both time‐resolved angiograms and quantitative perfusion images noninvasively and without the use of a contrast agent. Conventionally, angiograms, perfusion maps, and structural images are acquired separately. This can be time‐consuming, particularly if high spatial and/or temporal resolution is required, which can make acquiring all of these modalities within a busy clinical protocol infeasible, leaving the clinician with incomplete information.

A method was previously proposed to partially mitigate this issue: combined angiography and perfusion using radial imaging and ASL (CAPRIA).^3^, ^4^ This approach allows 4D time‐resolved angiographic and perfusion information to be acquired noninvasively from a single scan and has several advantages: (a) It is time‐efficient because both angiographic and perfusion images can be reconstructed from the same raw k‐space data; (b) the use of a golden ratio–based readout allows the spatial and temporal resolution of each reconstruction to be chosen retrospectively; (c) it does not suffer from significant signal dropout or distortion artifacts common in ASL perfusion imaging; and (d) spatiotemporal correlations can be leveraged to improve image quality in reconstruction.

However, CAPRIA also has some drawbacks: (a) Structural images have to be acquired separately, increasing the total scan time; (b) background suppression of static tissue signal is limited, leading to noise amplification, particularly later in the readout during the lower SNR perfusion phase; (c) the use of nonselective excitation pulses means a large FOV has to be used in reconstruction, increasing the computational burden, as well as including the ASL labeling plane in the reconstructed image, which adds considerable aliased signal from static tissue perturbations due to the labeling process itself; and (d) the resulting images were qualitative only.

In this work, we propose an extension of CAPRIA to resolve these issues: CAPRIA with structural contrast (CAPRIA+S). We aim to be able to reconstruct all three modalities from a single 6 min scan, maximizing scan efficiency and ensuring perfect co‐registration of all the data. In addition, we present models and postprocessing strategies to allow quantitative metrics to be extracted from all three modalities and perform initial comparisons of CAPRIA+S with time‐ and resolution‐matched conventional angiography and ASL perfusion imaging, with additional T_1_‐weighted structural imaging. This builds upon work previously presented in abstract form.^5^

## 2 METHODS

### 2.1 Pulse sequence design

**FIGURE 1:**
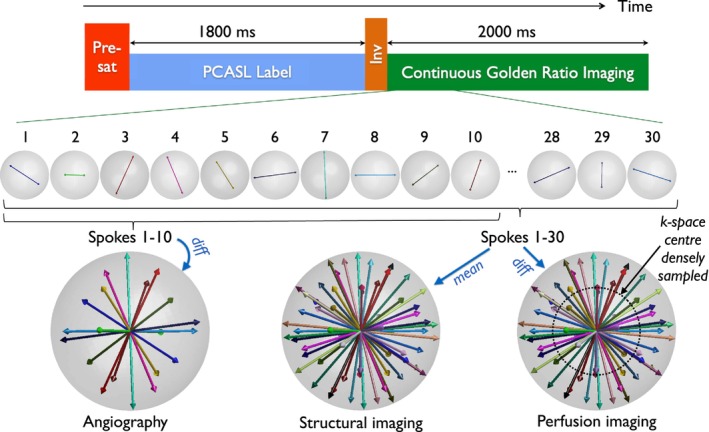
Schematic of the CAPRIA+S pulse sequence. After a pre‐saturation (“Pre‐sat”) module and PCASL labeling pulse train, an inversion (“Inv”) pulse is used to reduce the average tissue signal during the long continuous golden ratio 3D radial readout period, as well as providing time‐varying T_1_‐weighted contrast. The golden ratio‐based readout means that the raw k‐space data can be reconstructed into many time‐resolved images at different spatial and temporal resolutions for different purposes: difference (“diff”) data between label and control can be reconstructed at high spatial and temporal resolution for angiography using a small temporal window, or lower temporal and spatial resolution (using just the more densely sampled center of k‐space) for perfusion imaging using a larger temporal window. In addition, the mean label/control signal can be taken to retain the static tissue, allowing T_1_‐weighted structural images to be reconstructed at high spatial resolution and low temporal resolution from the same raw dataset. The temporal window used for reconstruction can be arbitrarily shifted across the readout to generate many images at different timepoints after the preparation phase. To achieve sufficient k‐space sampling, data are combined across many repeats of this process, as illustrated in Figure S1. CAPRIA+S, combined angiography and perfusion using radial imaging and arterial spin labeling with structural contrast; diff, difference; Inv, inversion; PCASL, pseudo‐continuous ASL; Pre‐sat, pre‐saturation.

A schematic of the CAPRIA+S pulse sequence is shown in Figure 1. It shares some key features with the original CAPRIA approach, including a pre‐saturation module to remove spin‐history effects and provide some background suppression, a pseudo‐continuous ASL (PCASL)^6^ pulse train to label blood water flowing into the brain, and a continuous 3D golden ratio^7^ radial (“koosh ball”) spoiled gradient echo readout, which can be retrospectively segmented into temporal frames using an arbitrary number of radial spokes, allowing time‐resolved images to be reconstructed with flexible spatial and temporal resolution. The subtraction of PCASL label and control data isolates the blood signal; thus, for example, a small number of radial spokes can be grouped to reconstruct angiographic images with high spatial and temporal resolution but with a high undersampling factor that can be tolerated with this high SNR and sparse signal. Perfusion images can be reconstructed with higher sensitivity by grouping a larger number of radial spokes (lower temporal resolution) using only the central region of k‐space where the sampling is denser, improving the conditioning of the reconstruction.

**FIGURE 2:**
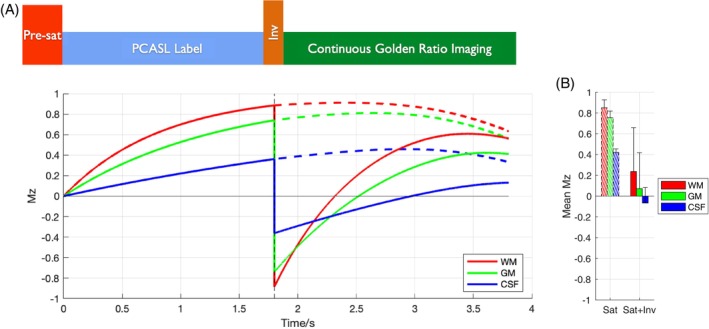
Tissue signal simulations: After the *M*_*z*_ is set to zero by the Pre‐sat module, different tissues within the imaging region (WM, GM, and CSF; see legend) recover at different rates during the PCASL pulse train (A). In the original CAPRIA implementation, no additional inversion (“Inv”) pulse was used (Sat only), leading to high tissue signals during the readout period (dashed lines). The additional inversion pulse in CAPRIA+S (Sat + Inv) reduces the average *M*_*z*_ during the readout, as shown in (B) but also introduces time‐varying T_1_‐weighted contrast during the readout, allowing multiple structural images with different T_1_‐weightings to be reconstructed from the raw k‐space data. Note that the *M*_*z*_ evolution during the readout period includes the combined effects of T_1_ recovery and the variable flip angle readout excitation pulses. CSF, cerebrospinal fluid; GM, gray matter; Inv, inversion; *M*_*z*_, longitudinal magnetization; Sat, saturation; WM, white matter.

However, for CAPRIA+S, a frequency offset corrected inversion (FOCI) pulse (as used in previous studies^8^) has been added immediately after the PCASL pulse train. This both improves background suppression, reducing the average static tissue signal during the readout to better suppress physiological noise, while also resulting in time‐varying T_1_‐weighted contrast in the static tissue signal, as shown in Figure 2, without affecting the magnitude of the control‐label blood difference signal (see Figure S2). Rather than only taking the *difference* between label and control data to isolate the blood signal, the *mean* signal can also be used for image reconstruction, retaining the static tissue signal and allowing images with different T_1_‐weightings to be produced at different effective TIs within the long golden ratio readout. With this approach, angiographic, perfusion, and structural images can all therefore be reconstructed from a single raw k‐space dataset.

A number of additional modifications have been made to allow to improve efficiency and to overcome some of the other drawbacks of CAPRIA mentioned above: (a) The PCASL labeling duration has been extended to 1800 ms to boost the labeled blood signal and bring this approach into line with consensus paper recommendations^9^; (b) slab‐selective excitation pulses are used during the readout so that a reduced FOV can be reconstructed in the inferior‐superior direction, lowering the computational burden and excluding aliased static tissue signal from the labeling plane, which can be problematic in these heavily undersampled scans; and (c) the radial spoke direction increments according to the golden ratio *first* across repeated label/control pairs of ASL preparations and *then* across the readout, as previously proposed,^10^, ^11^ improving the k‐space sampling efficiency for any desired temporal resolution (illustrated in Figure S1).

### 2.2 Subjects and scan protocol

In order to demonstrate the image quality achievable with CAPRIA+S and compare it to conventional methods, seven healthy volunteers (two female, age range 23–44) were scanned under a technical development protocol agreed by local ethics and institutional committees on a 3 T Prisma scanner (Siemens Healthineers, Erlangen, Germany) using a 32‐channel head coil.

A quick (1 min 22 s) time‐of‐flight (TOF) angiogram was acquired in the neck to enable positioning of the PCASL labeling plane.^12^ This was followed by a CAPRIA+S protocol (˜6 min), and spatial resolution‐ and total time‐matched conventional 3D multi‐slab TOF angiography (˜3 min) and four‐shot segmented 3D‐gradient and spin echo (GRASE)^13^ PCASL perfusion imaging, both multi‐postlabeling delay (PLD) and a single PLD, as suggested by the ASL white paper (WP),^9^ (each 2 min 30 s + 30 s for a calibration (M0) image). In this way, the total time for conventional angiography and one of the perfusion protocols was also approximately 6 min, matching the CAPRIA+S scan time. An additional rapid T_1_‐weighted MPRAGE^14^ scan was also acquired (3 min 40s) for comparison with the CAPRIA+S structural images and an extra 3D‐GRASE calibration image (30s) with opposed phase‐encoding (left–right rather than right–left) was also acquired to allow for distortion correction of the 3D‐GRASE data.

Spatial and temporal resolution were matched as closely as possible between CAPRIA+S and the comparison protocols, including 1.1 mm isotropic resolution for angiography and 3.4 mm isotropic resolution for perfusion imaging, with six perfusion PLDs ranging from ˜175 ms up to ˜1800 ms (with 1800 ms only for the WP 3D‐GRASE protocol). Due to the short time available for the comparison protocols, some compromises had to be made, including a reduced FOV relative to CAPRIA+S, the use of high parallel imaging (GRAPPA^15^) acceleration factors for TOF angiography, and the use of poorer spatial resolution (1.7 mm isotropic) for the MPRAGE (compared to 1.1 mm isotropic for CAPRIA+S structural images). Full details of the protocol parameters are given in Table 1.

**TABLE 1:** Imaging and reconstruction parameters.

	Parameter	CAPRIA+S			Conventional			
		Angiography	Perfusion	Structural	TOF	3D‐GRASE MPLD	3D‐GRASE SPLD (WP)	MPRAGE
ASL	Labeling duration (ms)	1800			*–*	1800		*–*
	Postlabel delay (ms)	Chosen during reconstruction^a^			–	175, 500, 825, 1150, 1475, 1800	1800	–
	Background suppression	WET pre‐saturation + single inversion pulse			–	WET pre‐saturation + double inversion pulses		–
Readout	Acquired matrix size	176 × 176 × 176			176 × 150 × 120^b^	64 × 51 × 36		128 × 116 × 128
	Nominal voxel size (mm^3^)	1.1 × 1.1 × 1.1			1.1 × 1.1 × 1.1	3.4 × 3.4 × 3.4		1.7 × 1.7 × 1.7
	Flip angle (°)	2–9 (quadratic)			18	90 (excite), 120 (refocus)		8
	TE (ms)	4.74			3.42	31		3.71
	TR (ms)	9.1			21.0	Variable	3950	8.8
	Bandwidth (Hz/Pixel)	144			186	2298		200
	Imaging time after labeling (ms)	1963			–	196		–
	Partial Fourier factor	–			0.74 (readout)	–		–
	Spokes/lines per ASL preparation	216			–	459 (4 segments)		–
	Label/control pairs	48			–	1 per PLD	5	–
	Acquisition time (min:sec)	6:10			3:04	2:30 + 0:30 (M0)	2:38 + 0:30 (M0)	3:40
Reconstruction	Temporal resolution (ms)	164	327	327	*–*	˜325	*–*	*–*
	Time frames	12	6	6	*–*	6	1	*–*
	Spatial resolution (mm^3^)	1.1 × 1.1 × 1.1	3.4 × 3.4 × 3.4	1.1 × 1.1 × 1.1	*–*	3.4 × 3.4 × 3.4		1.7 × 1.7 × 1.7
	Total spokes/lines per frame	864	1728	1728	*–*	1836		*–*
	Undersampling factor	56.3	3.0	28.2	4.0	1.0		1.0
	LLR regularization factor	7 × 10^−2^	10^−1^	10^−1^	*–*	*–*		*–*
	LLR patch size (voxels × frames)	15 × 15 × 12	7 × 7 × 6	5 × 5 × 6	*–*	*–*		*–*
	LLR iterations	200	200	200	*–*	*–*		*–*
	Reconstruction time (h)	9.6	0.35	4.8	*–*	*–*		*–*

*Note*: Note that only the CAPRIA+S parameters for protocols B/C are shown here for brevity. Protocol A only differed in the use of 47 label‐control pairs rather than 48, with minor changes to scan time (6 min 2 s vs. 6 min 10 s) and acceleration factors.

^a^ Different postlabeling delays are achieved by reconstructing images at different times after the PCASL pulse train. Minimum PLD = 33 ms + ½ × temporal resolution. Maximum PLD = 33 ms + (time frames – ½) × temporal resolution.

^b^ Acquired in six slabs.

Abbreviations: ASL, arterial spin labeling; CAPRIA+S, combined angiography and perfusion using radial imaging and arterial spin labeling with structural contrast; GRASE, gradient and spin echo; LLR, locally low rank; MPLD, multi‐postlabeling delay; PCASL, pseudo‐continuous ASL; PLD, postlabeling delay; SPLD, single postlabeling delay; TOF, time of flight; WET, water suppression enhanced through T1 effects; WP, white paper.

The subjects were scanned with one of three protocols: protocol A (the first four subjects) used a CAPRIA+S scan with 47 ASL label/control pairs (6 min 2 s), whereas protocols B (subjects 5 and 6) and C (subject 7) used 48 ASL label/control pairs (6 min 10s), to evaluate whether the resulting change in the golden ratio ordering affects an artifact seen in preliminary structural CAPRIA+S reconstructions. Protocols A and B included repeat CAPRIA+S and 3D‐GRASE perfusion scans to allow the assessment of between‐scan repeatability, whereas protocol C used only a single measurement to confirm the CAPRIA+S structural image quality.

### 2.3 Image reconstruction

Locally low rank (LLR) reconstructions are particularly well suited to CAPRIA/CAPRIA+S data,^4^ minimizing signal aliasing and noise amplification while improving spatial resolution by leveraging spatiotemporal correlations and the variable k‐space sampling patterns across time. This is particularly important for this highly undersampled data, with acceleration factors of 56, 3, and 28 for angiographic, perfusion, and structural reconstructions, respectively. The LLR approach,^16^ utilizing cycle spinning^17^ and the proximal optimized gradient method,^18^ was therefore applied to reconstruct angiographic, perfusion, and structural images separately from each raw CAPRIA+S dataset. Regularization factors and patch sizes were optimized empirically for angiography, perfusion, and structural imaging separately and are given in Table 1.

Coil sensitivities were estimated with the adaptive combine algorithm^19^ and compressed to eight virtual coils^20^ to reduce the computational burden. Reconstruction was performed on the Oxford Centre for Functional MRI of the Brain (FMRIB) compute cluster (equipped with EPYC 7643 2.3GHz CPUs from AMD, Santa Clara, USA) using four CPUs, taking between ˜10 hours for the high spatiotemporal resolution angiographic reconstructions down to ˜20 min for the perfusion reconstructions (see Table 1).

### 2.4 Signal modeling

#### 2.4.1 Angiographic modeling

In order to extract physiological parameters from the CAPRIA+S dynamic angiograms, we start with the kinetic model for PCASL angiography^21^ and incorporate a modification to account for the variable flip angle excitation pulses used during the readout.^4^ For this study, because the imaging region is positioned just above the labeling plane, it can be assumed that all of the labeled blood experiences all of the RF excitation pulses, but we keep the effects of dispersion to allow model fitting to real data, leaving us with the angiographic signal:

(1) $$S_{\text{angio}}\left(r,t_{i}\right)=S_{0}v\left(r\right)\sin\alpha_{i}R\left(t_{i}\right)\int_{t_{i}-\delta_{t}\left(r\right)-\tau }^{t_{i}-\delta_{t}\left(r\right)}\exp\left(-\frac{\delta_{t}\left(r\right)+t_{d}}{T_{1b}}\right)D\left(r,t_{d}\right)dt_{d},$$

where **r** is the voxel location, *t*_*i*_ is the time of the *i* ^*th*^ RF pulse relative to the start of PCASL labeling, *S*_0_ is a scaling factor, *v* is the volume of blood within arteries in that voxel, *α*_*i*_ is the flip angle of the *i* ^th^ RF excitation pulse, *δ*_*t*_ is the time taken for blood to travel from the labeling plane to the voxel, T_1b_ is the longitudinal relaxation time of blood, *t*_*d*_ is an additional time delay due to dispersion, *D* is the dispersion kernel, and *R* is the additional attenuation of the ASL difference signal due to previous RF pulses, given by:

(2) $$R_{i}=\left\{\begin{array}{ll} 1 & i=1\\ \prod_{j=1}^{i-1}\cos\alpha_{j} & i>1 \end{array}\right..$$

The dispersion kernel, *D*, has been shown to be well modeled by a gamma variate function^21^, ^22^ using a parameterization similar to Rausch et al.^23^:

(3) $$D\left(t_{d},p\left(r\right),s\left(r\right)\right)=\frac{s}{\Gamma \left(1+\mathrm{ps}\right)}e^{-st_{d}}{\left(st_{d}\right)}^{\mathrm{ps}},$$

where Γ is the gamma function, which can be rapidly evaluated numerically:

(4) $$\Gamma \left(z\right)=\int_{0}^{\infty }x^{z-1}e^{-x}\mathrm{dx}.$$

The integral in Equation ((1) can be evaluated numerically,^21^ but a faster and more robust solution is possible using the incomplete gamma function, Γ_inc_, which can be rapidly evaluated in many software packages, yielding a simplified form:

(5) $$\begin{array}{cc} S_{\text{angio}} & =S_{0}v\sin\alpha_{i}Re^{\delta_{t}/T_{1b}}\left[\Gamma_{\mathrm{inc}}\left(\left(s+1/T_{1b}\right)\left(t_{i}-\delta_{t}\right),1+\mathrm{ps}\right)\right.\\ & \;\left.-\;\Gamma_{\mathrm{inc}}\left(\left(s+1/T_{1b}\right)\left(t_{i}-\delta_{t}-\tau \right),1+\mathrm{ps}\right)\right], \end{array}$$

where:

(6) $$\Gamma_{\mathrm{inc}}\left(x^{\prime },z\right)=\int_{0}^{x^{\prime }}x^{z-1}e^{-x}\mathrm{dx}.$$

This approach was incorporated in the variational Bayesian model fitting package, FABBER,^24^ part of FMRIB Software Library (FSL).^25^ The modified code can be found at: https://github.com/tomokell/fabber_models_asl/tree/pcasldisp.

This model fitting procedure was applied to CAPRIA+S angiographic data within a vessel mask defined by taking the temporal maximum intensity projection of the angiogram; multiplying by a brain mask (obtained by applying BET^26^ to a CAPRIA+S structural image); thresholding at an empirically chosen value to retain most of the vessels; clustering and retaining only the two largest clusters (typically the anterior and posterior circulation), to remove noisy voxels. The signal scaling parameter, which is proportional to blood volume (*v*), was further divided by the bias field derived from the 3D‐GRASE perfusion data (described in section 2.5 below) to correct for receive coil nonuniformity.

#### 2.4.2 Perfusion modeling

The CAPRIA+S perfusion signal can be modeled as^4^:

(7) $$S_{\text{perf}}=\Delta M_{B,i}R\sin\alpha_{i},$$

where $\Delta M_{B,i}$ is the Buxton model for the (P)CASL perfusion signal.^27^ This modification was also made to the FABBER method, allowing CAPRIA+S perfusion data to be quantified using the BASIL pipeline,^28^ which calls FABBER internally. To compare more directly to the single‐PLD (WP) 3D‐GRASE data, the final CAPRIA+S perfusion image at the longest PLD (i.e., the final frame of the readout) was also isolated and used to quantify cerebral blood flow (CBF) on its own.

3D‐GRASE perfusion images were processed in a similar manner, with additional intervolume motion correction and distortion correction using the blip‐reversed *M*_0_ images via the FSL tool *topup*.^29^

#### 2.4.3 Structural (static tissue) modeling

The static tissue signal changes during the CAPRIA+S pulse sequence can be derived from simple T_1_ decay and RF attenuation considerations, allowing examination of the signal behavior (e.g., for predicting the T_1_‐weighted contrast at different timepoints) and fitting to real data to extract quantitative estimates of tissue T_1_. Here, we assume perfect spoiling and a short‐enough TE to neglect T_2_ decay. After the pre‐saturation module, we assume perfect saturation, followed by T_1_ recovery during the PCASL pulse train until just before the center of the inversion pulse at time *t* = *τ*_inv_^−^, which inverts the magnetization just after the inversion pulse at *t* = *τ*_inv_^+^ with efficiency α_inv_:

(8) $$M_{z}=\left\{\begin{array}{ll} 0 & t=0\\ 1-e^{-\tau_{\mathrm{inv}}/T_{1}} & t={\tau_{\mathrm{inv}}}^{-}\\ \left(1-e^{-\tau_{\mathrm{inv}}/T_{1}}\right)\left(1-2\alpha_{\mathrm{inv}}\right) & t={\tau_{\mathrm{inv}}}^{+} \end{array}\right.,$$

where here the equilibrium magnetization, *M*_0_, is set to be 1 for brevity. Further T_1_ recovery occurs until just before the first excitation pulse at *t* = *t*_0_:

(9) $${M_{z,1}}^{-}=1-\left(1-M_{z}\left(t={\tau_{\mathrm{inv}}}^{+}\right)\right)e^{-\left(t_{0}-\tau_{\mathrm{inv}}\right)/T_{1}}$$

Then, the transverse magnetization, *M*_*xy,i*_; the longitudinal magnetization just after the RF pulse, *M*_*z,i*_^+^; and the longitudinal magnetization just before the next excitation pulse, *M*_*z,i*+1_^−^ at the *i* ^th^ TR period, can be calculated iteratively as:

(10) $$\begin{array}{cc} M_{\mathrm{xy},i} & ={M_{z,i}}^{-}\sin\alpha_{i}\\ {M_{z,i}}^{+} & ={M_{z,i}}^{-}\cos\alpha_{i}\\ {M_{z,i+1}}^{-} & =1-\left(1-{M_{z,i}}^{+}\right)e^{-\mathrm{TR}/T_{1}}. \end{array}$$

The calculated CAPRIA+S signal at each timepoint, *M*_*xy,i*_, can then be averaged over the time period of each structural image frame and scaled by an additional factor of *M*_0_.

To fit this model to the CAPRIA+S structural images so that quantitative estimates of tissue T_1_ could be derived, the data were first phase corrected by multiplying through by exp(*−iϕ*), where *ϕ* is the phase in each voxel of the last frame, when the magnetization has recovered to a positive *M*_*z*_ state, before the real part of the signal is taken. The MatLab fitting routine *fmincon* (v2024b, Mathworks, Natick, MA) was then used to fit for *M*_0_, *α*_inv_, T_1_, and a relative *B*_1_^+^ scaling factor, *B*_1,rel_^+^ (to account for transmit inhomogeneity), in each voxel by minimizing the sum of squared differences between the phase‐corrected real CAPRIA+S signal and the model fit. Parameters were constrained to the ranges *M*_0_ ∈ [0, 10^10^], *α*_inv_ ∈ [0.5, 1], T_1_ ∈ [0.1, 10] s, *B*_1,rel_^+^ ∈ [0.9, 1.1], and initialized with fits to low spatial resolution (3.4 mm isotropic) data first, which was found to produce more robust results in preliminary testing.

### 2.5 Perfusion calibration procedure

In order to obtain perfusion measurements in absolute units, a measure of the equilibrium magnetization of blood, *M*_0b_, is required. In conventional PCASL imaging, this is normally achieved by acquiring a separate scan without ASL labeling or background suppression using a long TR to ensure the magnetization is close to equilibrium. For CAPRIA+S, however, data acquired across all ASL preparations are used to reconstruct the final images, so producing a calibration scan in the same manner would take as long as the CAPRIA+S acquisition itself. To avoid the need for any additional scan time, we propose to calibrate the signal using the same raw dataset, as follows.

CAPRIA+S structural images are reconstructed at the same spatial resolution as the perfusion data (3.4 mm isotropic). FSL's FAST (FMRIB's automated segmentation tool)^30^ is applied to the temporal average data to estimate and correct for the combined transmit and receive bias field. A white matter (WM) partial volume map derived from applying FSL's *fsl_anat* to the MPRAGE image is linearly registered to the CAPRIA+S structural images and thresholded at 0.9 to generate a WM mask. The low‐resolution CAPRIA+S structural images are then phase corrected and the real part taken (as described in section 2.4.3) before the mean signal in the WM mask is taken. The CAPRIA+S structural model described above is then fit to this curve to extract an estimate of the equilibrium magnetization of WM. This is converted into an estimate of *M*_*0b*_ by dividing by the partition coefficient for WM (assumed to be 0.82 here).

The CAPRIA+S perfusion data are then prepared for model fitting by phase correcting (using the low‐resolution structural images as a reference); taking the real part; multiplying by −1 (to accommodate the single inversion pulse); and then dividing through by the bias field, *M*_*0b*_, and the PCASL inversion efficiency (assumed to be 0.85).

Two calibration approaches were used for the 3D‐GRASE data: The multi‐postlabeling delay data were bias field corrected (using the ratio of *M*_0_ images with and without the vendor provided “pre‐scan normalize” option, which corrects for receive coil nonuniformity using a rapid pre‐scan procedure), and then a WM region of interest was used as the reference region for the BASIL pipeline to mimic as closely as possible the CAPRIA+S calibration approach. For the single PLD 3D‐GRASE data, the WP^9^‐recommended voxelwise calibration approach was taken.

### 2.6 Image quality assessment

Because the conventional angiography (TOF) and structural imaging (MPRAGE) protocols provide qualitative data only and spatial resolution was lower than CAPRIA+S for the structural imaging modality, only qualitative comparisons were performed. For the quantitative perfusion data, mean gray matter (GM) perfusion was extracted and scan–rescan repeatability was calculated as the spatial correlation (*R* ^2^) between the repeated scans using CBF maps linearly registered^31^ to the MPRAGE image to ensure alignment. Differences in mean GM CBF and repeatability across the four methods (multi‐PLD/single‐PLD CAPRIA+S and multi‐PLD/single‐PLD 3D‐GRASE) were analyzed using a multi‐way analysis of variance, with imaging method and subject as factors. Where significant effects were found, post hoc paired *t*‐tests were used to assess differences between pairs of methods.

## 3 RESULTS

**FIGURE 3:**
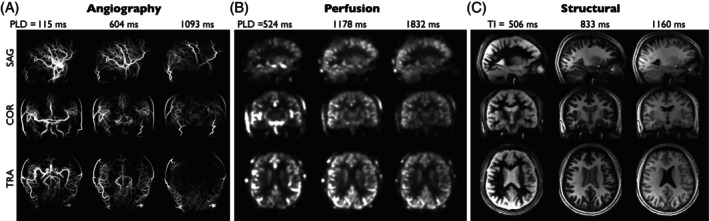
Example angiography, perfusion, and structural images reconstructed from a single raw CAPRIA+S k‐space dataset (protocol B) acquired in 6 min: (A) MIPs of three selected angiographic frames in SAG, COR, and TRA views reconstructed with high spatiotemporal resolution, showing the flow of labeled blood through the vascular tree. Note that the use of a long PCASL labeling duration means that the first frame shows much of the vasculature filled with labeled blood. (B) Example slices and frames from perfusion images reconstructed at lower spatiotemporal resolution to improve sensitivity, demonstrating the expected patterns of macrovascular signal at early PLDs, followed by exchange of labeled blood water into the tissue at later PLDs. (C) T_1_‐weighted structural images reconstructed from the average of label and control data at selected times after the inversion pulse (TIs) at high spatial and low temporal resolution, demonstrating the expected contrast changes, including nulling of certain tissues at particular TIs. All frames can be viewed in videos [Link], [Link], and S3, and a more detailed inspection of each modality can be found in Figures 4, 6, and 8. COR, coronal; MIP, maximum intensity projections; PLD, postlabeling delay; SAG, sagittal; TRA, transverse.

Figure 3 shows example 4D angiographic, perfusion, and structural images reconstructed from a single raw CAPRIA+S dataset acquired in 6 min. All frames of these images can be viewed in videos [Link], [Link], and S3. Good image quality is observed in all three modalities, clearly showing the blood flowing through the vasculature and perfusing the tissue, with good delineations of brain anatomy and different tissue types with multiple contrasts. Comparison of data‐acquired protocols A and B/C shows care needs to be taken in choosing the number of ASL preparations in the CAPRIA+S protocol to avoid structural artifacts due to a complex interaction between spoiling and the golden ratio ordering approach, although angiographic and perfusion images appear to be insensitive to this choice (see Figures S3 and S4).

**FIGURE 4:**
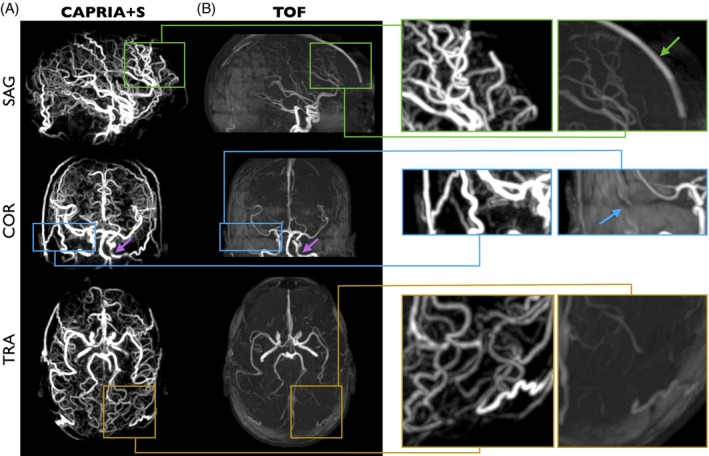
Comparison of CAPRIA+S angiography with conventional TOF angiography: (A) CAPRIA+S temporal maximum intensity projection across the first seven angiographic frames, shown in different views. (B) TOF maximum intensity projections after correction for the nonuniform coil receive sensitivity to reduce overlying tissue interference as far as possible. Zoomed sections and arrows demonstrate the lack of venous contamination signal in CAPRIA+S that is present in TOF (green); TOF slab boundary artifacts, which interrupt vessel signal in slower flowing regions (blue); and the greatly improved visibility of smaller, distal vessels in CAPRIA+S (yellow). There is some apparent signal reduction in proximal vessels (purple arrows), which is present in both TOF and CAPRIA+S images, perhaps due to flow‐ and/or *B*_0_‐based dephasing. TOF, time of flight.

**FIGURE 5:**
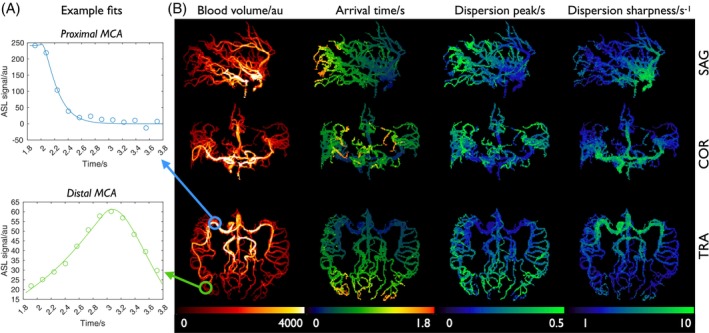
Angiographic model fitting results: (A) example fits of the dynamic CAPRIA+S data to the kinetic model in voxels within the proximal (blue, top) and distal (green, bottom) MCA. (B) Maximum intensity projections of the resulting parameter maps, showing the locations of the voxels used in (A) and demonstrating the expected patterns of longer blood arrival time and increased dispersion (delayed peak and lower sharpness) in distal vessels. In these maps, the scaling factor S_0_ is merged with the blood volume and estimated as a single scaling factor in arbitrary units. MCA, middle cerebral artery.

CAPRIA+S angiography has a number of advantages over the rapidly acquired conventional TOF protocol (Figure 4), including much clearer delineation of distal and slower flowing vessels (e.g., branches of the external carotid arteries), lack of venous contamination, and slab boundary artifacts, although a small residual loss of signal is observed in some proximal vessels, perhaps due to flow‐ and/or B_0_‐induced dephasing effects. An additional advantage of CAPRIA+S is the dynamic nature of the data, allowing visualization of flow through the vascular tree and fitting of a physiological model, as shown in Figure 5. Good fits to Equation (5) were observed, yielding parameter maps with the expected increases in both blood transit time and dispersion (longer time to peak, *p*; lower sharpness, *s*) moving toward more distal vessels.

**FIGURE 6:**
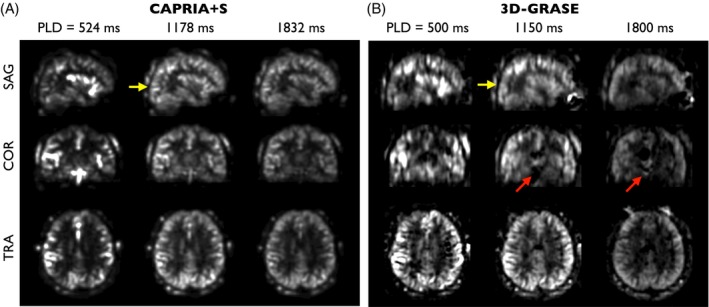
Qualitative comparison of CAPRIA+S perfusion images (A) with conventional 3D‐GRASE multi‐postlabeling delay data (B) at selected delay times. Comparable patterns of labeled blood flowing from the vessels into the tissue are seen in both datasets, although the macrovascular signal is more prominent in the CAPRIA+S data due to the lack of flow‐crushing spoiler gradients associated with the spin‐echo refocusing pulses. There may be some minor smoothing of the CAPRIA+S data due to the undersampled radial readout, but the 3D‐GRASE data appears to have lower SNR, increased through‐slice blurring (yellow arrows) and some additional artifact in inferior regions (red arrows). Note that the lower relative tissue signal in earlier CAPRIA+S frames is due to the use of a variable flip angle readout. GRASE, gradient and spin echo.

Dynamic perfusion images generated with CAPRIA+S also bear a close resemblance to multi‐PLD 3D‐GRASE perfusion images, as shown in Figure 6, with the expected patterns of macrovascular signal at early PLDs, followed by the labeled blood exchanging into the tissue at later PLDs. The macrovascular signal is less apparent in the 3D‐GRASE data, most likely due to the use of flow‐crushing refocusing pulses. Some of the artifacts seen in the 3D‐GRASE data, including through‐slice blurring and signal instability in inferior regions, do not appear in the CAPRIA+S images, which also have higher apparent SNR because there was only time for a single average at each PLD in the 3D‐GRASE protocol.

**FIGURE 7:**
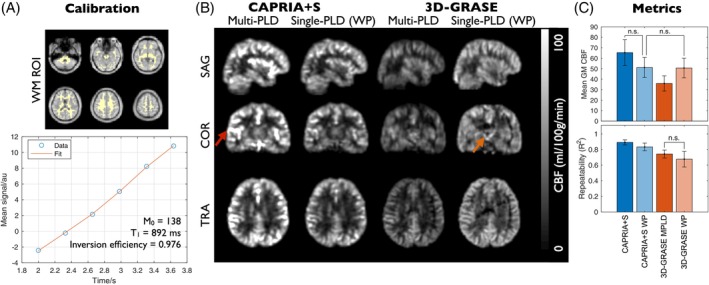
Quantitative perfusion comparison: (A) Example CAPRIA+S calibration procedure using a WM ROI. The phase‐corrected ROI average signal was fit to the static tissue model to extract estimates of the inversion pulse efficiency, tissue T_1_, and equilibrium magnetization (*M*_0_). Note that the near‐linear signal behavior results from the combination of T_1_ recovery with the amplification of signals at later time points from the variable flip angle readout. (B) Comparison of quantitative CBF maps between CAPRIA+S and 3D‐GRASE (both MPLD and single‐delay, WP methods). Comparable features are seen in these maps, although there may be some residual macrovascular contamination in the MPLD CAPRIA+S data (red arrow) and there are global CBF scaling differences. Some residual artifacts are visible in the 3D‐GRASE data (orange arrow). (C) Mean GM CBF metrics (top) reflect the observed global CBF differences across methods but show that single PLD CAPRIA+S gives comparable CBF estimates to single PLD 3D‐GRASE. Repeatability metrics (bottom) demonstrate that both CAPRIA+S approaches are significantly more repeatable than both 3D‐GRASE approaches. All differences are significant at *p* < 0.05 except those marked with n.s. CBF, cerebral blood flow; *M*_0_, equilibrium magnetization; MPLD, multi‐postlabeling delay; n.s., not significant; ROI, region of interest; WP, white paper.

Figure 7 shows the results of the quantitative perfusion comparison. The proposed CAPRIA+S calibration process appeared to work well, giving good fits to the WM average signal, which allowed CAPRIA+S perfusion maps to be produced in absolute units with the expected average GM values (around 60 mL/100 g/min). Absolute CBF maps showed similar features between the CAPRIA+S and 3D‐GRASE approaches, although some residual artifacts were still visible in the 3D‐GRASE data and there were absolute scaling differences depending on the method used for calibration, as has been noted previously.^32^ Some residual macrovascular contamination may be present in the multi‐PLD CAPRIA+S maps due to the lack of flow‐crushing spin‐echo pulses during the readout. This was less apparent in the single‐PLD (WP parameters) CAPRIA+S CBF maps, which were comparable to the 3D‐GRASE single‐PLD data, with no significant difference in the average GM CBF values. In addition, both CAPRIA+S approaches were significantly more repeatable than both the 3D‐GRASE methods, a marker of the improved signal stability.

**FIGURE 8:**
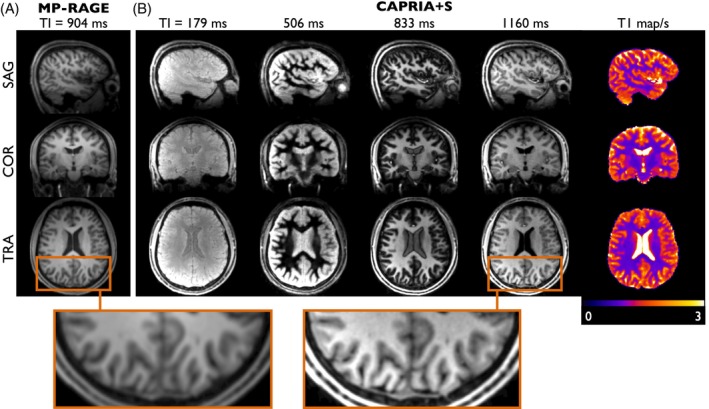
Comparison of conventional MPRAGE structural imaging (A) with multi‐contrast T_1_‐weighted CAPRIA+S images (B). CAPRIA+S structural images acquired at later TIs (e.g., TI = 1160 ms) show comparable tissue contrast and image details to conventional MPRAGE (see zoomed sections). However, CAPRIA+S also provides a range of other structural images, including those with certain tissues nulled (e.g., WM at TI = 506 ms). From this data, a quantitative T_1_ map can be derived (right column).

Finally, example CAPRIA+S structural images from protocol B are compared with conventional T_1_‐weighted MPRAGE imaging in Figure 8. The inability to match voxel sizes given the limited imaging time available for the MPRAGE protocol means a precise comparison is difficult, but comparable tissue contrast can be seen at later CAPRIA+S TIs. In addition, a variety of other T_1_‐weighted contrasts are produced, including those with some tissues close to their null time, which display even clearer boundaries between tissue types. The multi‐TI CAPRIA+S data can also be used to fit a quantitative T_1_ map, yielding T_1_ values in the expected ranges for GM/WM and CSF (see Figure 8B).

## 4 DISCUSSION

The proposed CAPRIA+S approach generates 4D angiograms, perfusion images, and multi‐contrast T_1_‐weighted structural images from a single 6 min scan that have significant advantages over conventional methods, which took considerably longer to acquire (˜10 min in total). CAPRIA+S angiograms show much clearer depiction of distal vessels, do not suffer from venous contamination or slab‐boundary artifacts, and are time‐resolved, giving additional hemodynamic information (e.g., for separating arterial and venous flow through an arteriovenous malformation) and allowing a kinetic model to be fit to the data, providing additional parameter maps that could be indicative of disease processes.^21^ CAPRIA+S perfusion images give comparable CBF maps to a conventional 3D‐GRASE approach but are more repeatable and do not suffer from through‐slice blurring or signal instability artifacts. In addition, CAPRIA+S structural images give comparable contrast to conventional MPRAGE, but the additional images reconstructed at other TIs provide additional information (e.g., with some tissues nulled), which could help with lesion identification and allow quantitative T_1_ maps to be estimated.

Similar advantages over conventional TOF for angiography have been noted previously with a similar CAPRIA protocol,^4^ although in this study a longer labeling duration and shorter scan time were used, greatly increasing the undersampling factor for CAPRIA+S, but this did not appear to have a detrimental effect on image quality. In addition, the TOF protocol was better matched in terms of spatial resolution, giving a fairer comparison. Despite this, the arteries do appear broader in CAPRIA+S angiograms compared to TOF, which could be because: (a) slower flowing blood at the vessel edges (resulting from laminar flow) will become quickly saturated during the TOF readout, reducing its signal intensity; (b) overlying static tissue obscures weaker signals at the vessel edges (due to slow flow and/or partial voluming) in TOF MIPs, which does not happen with ASL; or (c) some additional blurring could be present in the CAPRIA+S data due to the highly undersampled radial readout. This could be further explored in an angiographic phantom with well‐defined vessel sizes, or blurring could be mitigated through use of a more efficient 3D cones trajectory that better samples the periphery of k‐space.^33^

The only other disadvantage of CAPRIA+S for angiography is the slight loss of signal in some proximal vessels, most likely due to fast flow and/or B_0_‐related dephasing effects, although this could also be partly related to the rapid washout of the trailing edge of the bolus of labeled blood in these proximal vessels that are close to the labeling plane. Improvements could be seen by using a more proximal labeling plane (at a cost of increased T_1_ signal decay in distal regions) and/or the use of a readout with a shorter TE, such as the 3D‐cones approach,^34^, ^35^ which has been recently demonstrated to reduce proximal signal loss for CAPRIA^33^ and will be further explored for CAPRIA+S in future work.

Perfusion images produced with CAPRIA+S do not suffer from through‐slice blurring artifacts common in ASL perfusion images using 3D readouts that result from T_2_‐induced signal decay during the long readout train. Even though the readout duration was mitigated in this study using a four‐segment 3D‐GRASE acquisition, the blurring effect was still noticeable relative to CAPRIA+S. In addition, the use of segmented 3D‐GRASE readouts can lead to signal instabilities, particularly in inferior regions, likely arising from pulsatile motion or respiratory‐induced *B*_0_ field changes.

One notable difference between the modalities is the reduced macrovascular signal seen in 3D‐GRASE due to the use of spin‐echo refocusing pulses with larger crusher gradients on either side, which dephase magnetization in voxels with a range of flow velocities. This is likely one reason for the differences seen in quantitative CBF maps, particularly for the multi‐PLD CAPRIA+S data, so some further refinement in modeling dispersed macrovascular signal within the perfusion image time series is required in the future to mitigate this effect, perhaps leveraging the high SNR angiographic CAPRIA+S images that are intrinsically co‐registered to use as priors in the fitting process. Some global variation in the absolute CBF maps was also noted, most likely due to differences in the calibration procedures, which has been observed previously.^32^ This was particularly apparent in the lower CBF values obtained with the multi‐PLD 3D‐GRASE protocol, perhaps due to the combined use of a scanner receive coil sensitivity correction that does not accommodate B_1_^+^ effects and the use of a WM reference region, which is mostly concentrated toward the center of the head, leading to an overestimation of *M*_0,WM_ and therefore an underestimation of CBF. The ability to use the recommended voxelwise calibration approach^9^, ^32^ rather than a WM reference region method would likely benefit CAPRIA+S—and might be possible using the voxelwise M_0_ values derived from fitting the structural images. However, this warrants further investigation in future work.

Using only the final PLD CAPRIA+S data, however, led to CBF maps with comparable mean GM CBF values to the single‐PLD WP 3D‐GRASE protocol, showing that the proposed CAPRIA+S calibration approach was working well. In addition, CAPRIA+S was shown to be more repeatable than 3D‐GRASE, demonstrating that the increased imaging time available through efficiently acquiring multiple modalities simultaneously, as well as sampling the signal over a long readout and potential increases in signal stability arising from the frequent sampling of central k‐space, outweighed the lower signal available due to the use of smaller flip angles.

The ability to obtain perfectly co‐registered T_1_‐weighted structural images from the same acquisition is a considerable advantage of CAPRIA+S, and uses similar principles to the previously proposed MPnRAGE method.^36^ The golden ratio imaging approach allows for retrospective selection of the TI, which could accommodate images with specific tissues nulled, for example, that could enhance lesion detection or tissue segmentation, although further work is required to determine the optimal postprocessing strategies to best leverage this additional information and compare with resolution‐matched MPRAGE data, which was not possible in this study due to scan time constraints. In addition, it was demonstrated that quantitative estimates of tissue T_1_ can be obtained from fitting the multi‐TI CAPRIA+S data. Although most of the imaging region is quite far off‐resonance with respect to the PCASL pulses (˜25 kHz) and we did not observe any obvious artifacts, there is the potential for magnetization transfer effects to influence the structural images in inferior regions, which may need to be accounted for in quantitative modeling. Comparison with established T_1_ quantification methods also needs to be performed in future work, including how the use of a variable flip angle readout affects T_1_ estimation accuracy and whether it leads to greater sensitivity to B_1_^+^ inhomogeneity.

In addition to the ability to obtain structural images and quantitative information, the CAPRIA+S pulse sequence presented here benefits from a number of improvements relative to the original 4D CAPRIA method.^4^ These include better background suppression from the inversion pulse after the PCASL preparation (reducing physiological noise), the use of a slab‐selective excitation pulse that excludes the labeling plane (reducing aliased static tissue difference signal from the PCASL pulses and minimizing the required FOV for the reconstruction), and the use of a more efficient golden ratio ordering method proposed by Song et al.,^10^ ensuring perfect golden ratio ordering for all frames with any desired temporal resolution (maximizing k‐space sampling uniformity). However, this study also highlighted the need to carefully select the number of ASL preparations used in the protocol when using the Song et al.^10^ approach combined with spoiling along the readout direction to avoid accidental refocusing of magnetization from previous TRs. This did not appear to adversely affect the angiographic or perfusion reconstructions, perhaps because the angiographic signal is more rapidly dephased through flow‐related effects, making it less prominent in subsequent TRs; and the perfusion images used only the central portion of k‐space, away from the observed regions of refocused signal (Figure S4). Another strategy would be to rewind all gradient moments to zero and then use a spoiler gradient in a consistent direction at the end of each TR, although this would require additional time and reduce scan efficiency.

This study has a number of limitations. The use of a single inversion pulse provides useful T_1_‐weighted contrast for structural images but gives limited background suppression later in the readout, potentially leading to increased physiological noise. Additional inversion pulses during the readout or the use of time‐encoded preparations^37^, ^38^ to achieve a shorter readout could improve this, although structural image contrast may be compromised. In this study, we used only the LLR reconstruction approach and empirically optimized the reconstruction parameters, which were found to work well on the subjects investigated here but may need more careful tuning in other cohorts or with other protocols. Reconstruction times were also very long (many hours for angiography), which would hinder uptake in clinical practice. Refinement of the reconstruction algorithms and the use of GPU acceleration is an important further step toward making this approach clinically viable. Only healthy volunteers were investigated, but reoptimization of protocol parameters (e.g., the flip angle schedule) may be required for elderly or patient cohorts. In addition, the separate angiographic, perfusion, and structural imaging protocols used for comparison with CAPRIA+S represent conventional methods but did not incorporate state‐of‐the‐art acceleration and reconstruction methods such as compressed sensing^39^ or deep learning–based approaches,^40^ which would likely improve performance in the short imaging time available. A comparison with more advanced methods was beyond the scope of the current study but would be beneficial in future work.

The 3D golden ratio readout was found to work well here, but a similar strategy could be used with other readout schemes, including balanced steady‐state free precession^41^, ^42^, ^43^ readouts—or alternative trajectories such as stack‐of‐stars,^42^ multi‐slice golden angle radial methods,^11^ or 3D cones.^33^ Further improvements are also possible through recent developments such as subspace reconstructions^44^, ^45^ and motion correction,^46^ which will be explored in future work.

## 5 CONCLUSIONS

The proposed CAPRIA+S approach allows the generation of 4D angiographic, perfusion, and structural images in a single 6 min scan, with significant advantages in vessel visibility, reduced artifacts, increased signal stability, and time‐resolved information over separately acquired conventional acquisitions taking 10 min.

## CONFLICT OF INTEREST STATEMENT

t.w.o. is the sole author of a US patent relating to the CAPRIA method built upon in this work.

## Supporting information


**Figure S1** Sequence looping: a simple example showing a protocol which uses only two ASL preparations per PCASL condition, which are repeated in an interleaved manner, switching between label and control. The first five radial spokes acquired during the readout period are shown, with the number above each spoke showing the integer golden ratio counter used to determine the spoke orientation. Exactly the same spokes are acquired for label and control conditions as close together in time as possible, to minimize motion and drift artifacts. Using the ordering scheme of Song et al.,^10^ the golden ratio counter increments *down* the ASL preparations first, *then across the readout*. In this example, only two adjacent spokes are grouped to reconstruct each frame (so the temporal resolution is twice the TR), but this ordering approach ensures perfect golden ratio ordering within each frame. For example, frame 1 contains spokes 1 to 4 for both label and control conditions, wherease frame 2 contains spokes 5 to 8, and so on.
**Figure S2** Simulation of the blood signal during the CAPRIA+S pulse sequence, analogous to the static tissue simulations in Figure 2. In this case, the blood starts in the neck and therefore does not experience the pre‐saturation module, so blood in the control condition starts at equilibrium, and we assume the labeled blood is instantaneously inverted at the beginning of the PCASL pulse train for this visualization. If the inversion pulse (“Inv”) is applied (solid lines), the ASL control‐label contrast is inverted. However, if we assume a perfect inversion pulse, the magnitude of the signal difference is identical to the original CAPRIA approach which did not use an inversion pulse (dashed lines), as indicated by the black arrows.
**Figure S3** Golden ratio related structural artifacts: example angiographic (A), perfusion (B) and structural (C) CAPRIA+S images in the same subject acquired with 47 or 48 pairs of ASL preparations. Angiographic and perfusion images are almost indistinguishable, with no apparent artifacts, but the CAPRIA+S structural image reconstructed from the 47 ASL preparation scan has a wave‐like patterns (see zoomed section) suggestive of excess signal at higher spatial frequencies. These artifacts are not present in the 48 ASL preparations protocol. This artifact was consistently seen across the four subjects which used the 47 preparations protocol and was not present in all subjects that used the 48 preparations protocol (D), confirming this to be the source of the problem. This is likely due to interactions between the golden ratio ordering scheme and the gradient spoilers used in the acquisition, as explained further in Figure S4.
**Figure S4** Comparison of k‐space trajectories with the two CAPRIA+S protocols used in this study (with 47 or 48 pairs of label/control ASL preparations). The use of the Song et al.^10^ approach to golden ratio looping, which increments spoke angles with the golden ratio *first* down pairs of ASL preparations *and then* across the readout, means that consecutively acquired spokes in time can have many golden ratio increments between them. When 47 ASL preparations are used, this can result in adjacent spokes being close to anti‐parallel in some cases (spokes 10–12 acquired after ASL preparation 4 are shown in A). After each spoke, a spoiler gradient is added in the same direction as the readout gradient. To examine the effect of this spoiling scheme on magnetization excited by excitation pulse 10, the total trajectory of all three spokes with spoilers can be concatenated (as shown in B, with the gray shadows showing the projection of the total trajectory on to each orthogonal plane). This total trajectory loops back towards the k‐space centre during spoke 12, showing that magnetization excited by excitation pulse 10 will be largely refocused, leading to artefactual signal. In contrast, if 48 ASL preparations are used, the spoke distribution is improved, leading to previously excited magnetization being further dephased during the acquisition of the next spoke. This refocused signal can be seen in the acquired data with 47 ASL preparations (C, red arrow), which is avoided when 48 ASL preparations are used.

**Video S1** Video of the CAPRIA+S angiography maximum intensity projections from one subject shown in Figure 3.

**Video S2** Video of the CAPRIA+S perfusion images from one subject shown in Figure 3.

**Video S3** Video of the multi‐contrast CAPRIA+S structural images from one subject shown in Figure 3.

## Data Availability

CAPRIA+S image reconstruction, signal simulation, and model fitting code used in this study are openly available in GitHub (https://github.com/tomokell/capria_tools). Data underlying the plots and code to produce them, including statistical analysis, are openly available in Zenodo (https://zenodo.org/records/17087138). We are currently unable to share subject level data because of data protection issues, although our center is actively working on a solution to this.
