# Supplementary material for: Combined angiography and perfusion using radial imaging and arterial spin labeling with structural contrast
Source: Magn Reson Med. 2025 Sep 15;95(2):787–802. doi: 10.1002/mrm.70073 (PMC12681295; doi:10.1002/mrm.70073)
Supplement: Supplementary file 1 — Figure S1 Sequence looping: a simple example showing a protocol which uses only two ASL preparations per PCASL condition, which are repeated in an interleaved manner, switching between label and control. The first five radial spokes acquired during the readout period are shown, with the number above each spoke showing the integer golden ratio counter used to determine the spoke orientation. Exactly the same spokes are acquired for label and control conditions as close together in time as possible, to minimize motion and drift artifacts. Using the ordering scheme of Song et al., 10 the golden ratio counter increments down the ASL preparations first, then across the readout. In this example, only two adjacent spokes are grouped to reconstruct each frame (so the temporal resolution is twice the TR), but this ordering approach ensures perfect golden ratio ordering within each frame. For example, frame 1 contains spokes 1 to 4 for both label and control conditions, wherease frame 2 contains spokes 5 to 8, and so on. Figure S2 Simulation of the blood signal during the CAPRIA+S pulse sequence, analogous to the static tissue simulations in Figure 2. In this case, the blood starts in the neck and therefore does not experience the pre‐saturation module, so blood in the control condition starts at equilibrium, and we assume the labeled blood is instantaneously inverted at the beginning of the PCASL pulse train for this visualization. If the inversion pulse (“Inv”) is applied (solid lines), the ASL control‐label contrast is inverted. However, if we assume a perfect inversion pulse, the magnitude of the signal difference is identical to the original CAPRIA approach which did not use an inversion pulse (dashed lines), as indicated by the black arrows. Figure S3 Golden ratio related structural artifacts: example angiographic (A), perfusion (B) and structural (C) CAPRIA+S images in the same subject acquired with 47 or 48 pairs of ASL preparations. Angiographic and perfusion images [file MRM-95-787-s003.pdf]

Supporting Figures for "Combined Angiography and Perfusion using Radial Imaging and Arterial Spin Labeling with Structural Contrast"

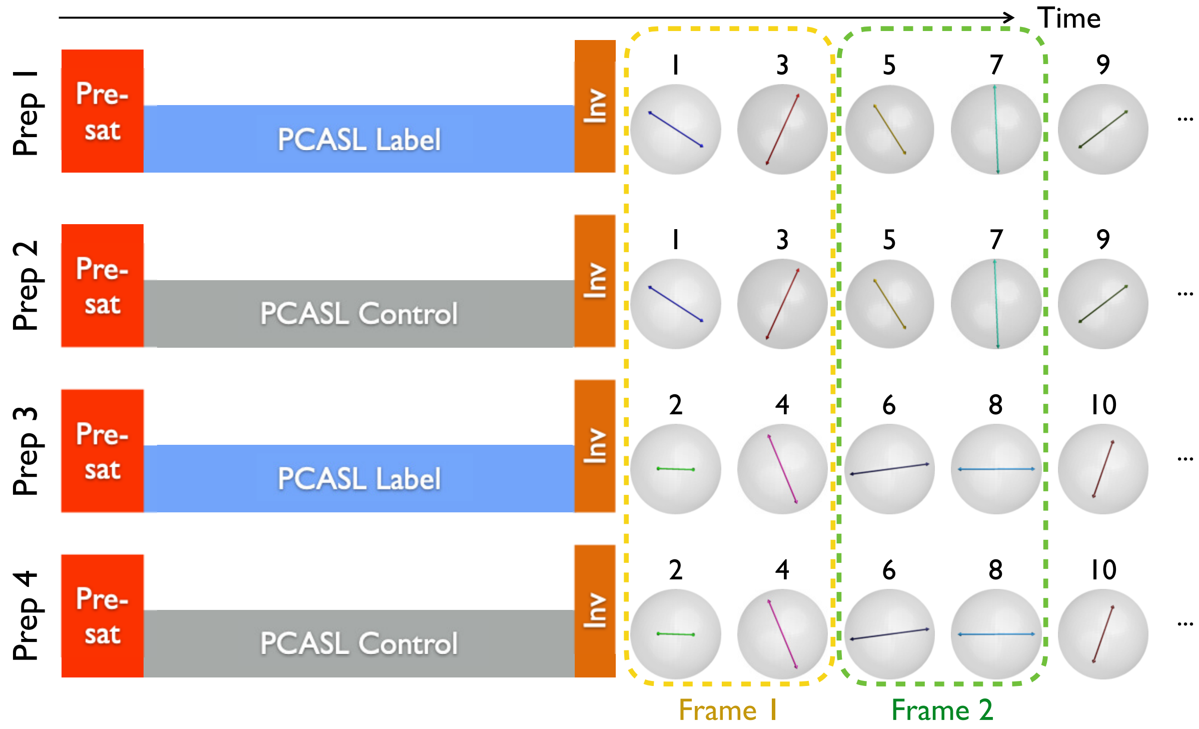

Supporting Figure S1: Sequence looping: a simple example showing a protocol which uses only two ASL preparations, which are repeated in an interleaved manner, switching between PCASL label and control modes. The first five radial spokes acquired during the readout period are shown, with the number above each spoke showing the integer golden ratio counter used to determine the spoke orientation. Exactly the same spokes are acquired for label and control conditions as close together in time as possible, to minimize motion and drift artefacts. Using the ordering scheme of Song et al.[10], the golden ratio counter increments *down* the ASL preparations first, *then across the readout*. In this example, only two adjacent spokes are grouped to reconstruct each frame (so the temporal resolution is twice the TR), but this ordering approach ensures perfect golden ratio ordering within each frame. For example, frame 1 contains spokes 1 to 4 for both label and control conditions, while frame 2 contains spokes 5 to 8, and so on.

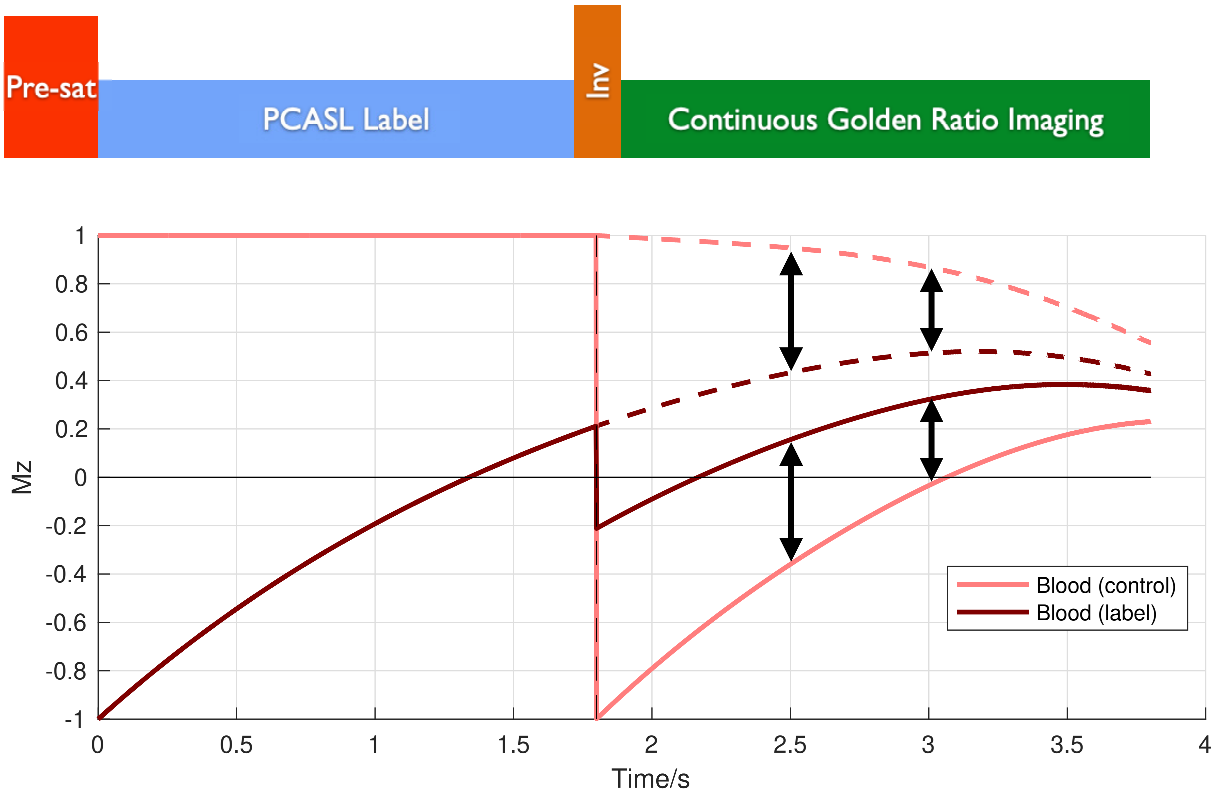

Supporting Figure S2: Simulation of the blood signal during the CAPRIA+S pulse sequence, analogous to the static tissue simulations in Figure 2. In this case, the blood starts in the neck and therefore does not experience the pre-saturation module, so blood in the control condition starts at equilibrium, and we assume the labeled blood is instantaneously inverted at the beginning of the PCASL pulse train for this visualization. If the inversion pulse ("Inv") is applied (solid lines), the ASL control-label contrast is inverted. However, if we assume a perfect inversion pulse, the magnitude of the signal difference is identical to the original CAPRIA approach which did not use an inversion pulse (dashed lines), as indicated by the black arrows.

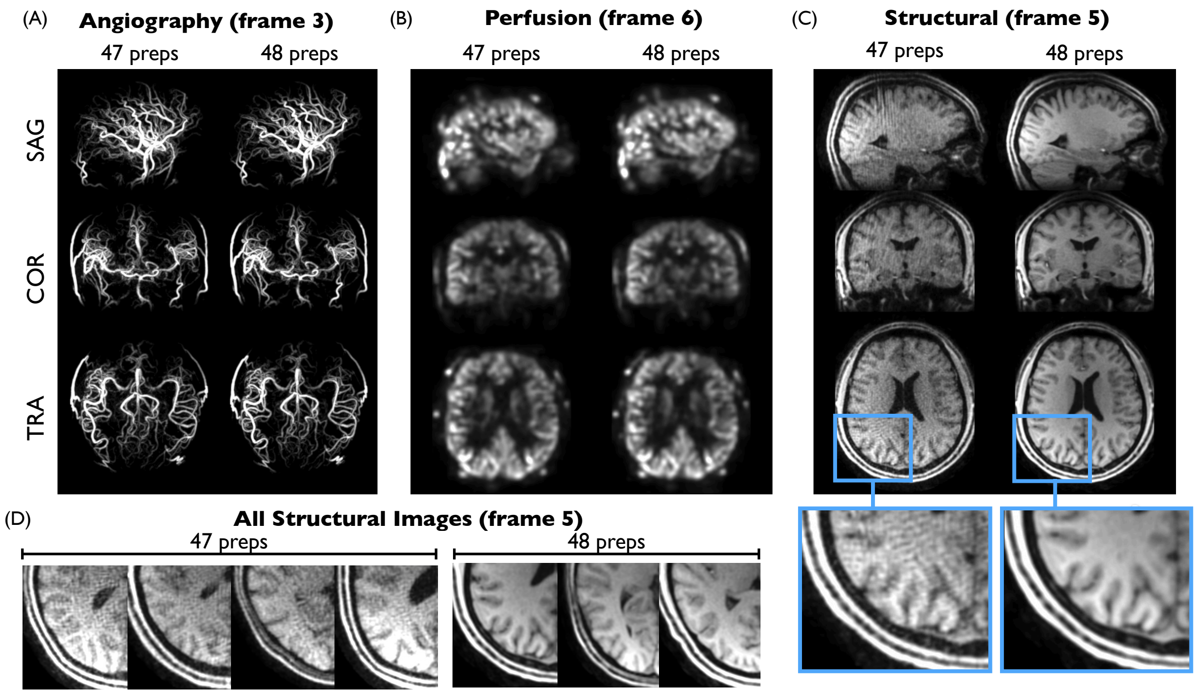

Supporting Figure S3: Golden ratio related structural artefacts: example angiographic (A), perfusion (B) and structural (C) CAPRIA+S images in the same subject acquired with 47 or 48 pairs of label/control ASL preparations. Angiographic and perfusion images are almost indistinguishable, with no apparent artefacts, but the CAPRIA+S structural image reconstructed from the 47 ASL preparation scan has a wave-like patterns (see zoomed section) suggestive of excess signal at higher spatial frequencies. These artefacts are not present in the 48 ASL preparations protocol. This artefact was consistently seen across the four subjects which used the 47 preparations protocol and was not present in all subjects that used the 48 preparations protocol (D), confirming this to be the source of the problem. This is likely due to interactions between the golden ratio ordering scheme and the gradient spoilers used in the acquisition, as explained further in Supporting Figure S4.

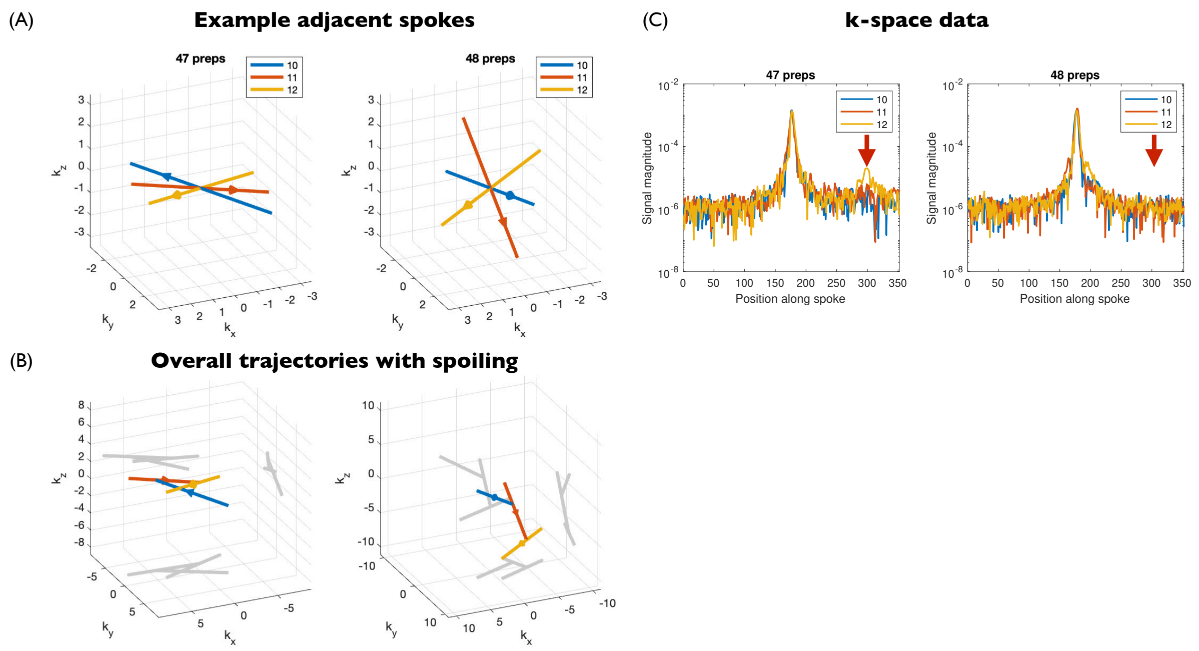

Supporting Figure S4: Comparison of k-space trajectories with the two CAPRIA+S protocols used in this study (with 47 or 48 pairs of label/control ASL preparations). The use of the Song et al.[10] approach to golden ratio looping, which increments spoke angles with the golden ratio *first* down repeats *and then* across the readout, means that consecutively acquired spokes in time can have many golden ratio increments between them. When 47 ASL preparations are used, this can result in adjacent spokes being close to anti-parallel in some cases (spokes 10-12 acquired after ASL preparation 4 are shown in A). After each spoke, a spoiler gradient is added in the same direction as the readout gradient, but if the total trajectory of all three spokes with spoilers are concatenated (B) it can be seen that magnetization excited by excitation pulse 10 can be largely refocused during the acquisition of spoke 12. In contrast, if 48 ASL preparations are used, the spoke distribution is improved, leading to previously excited magnetization being further dephased during the acquisition of the next spoke. This refocused signal can be seen in the acquired data with 47 ASL preparations (C, red arrow), which is avoided when 48 ASL preparations are used.
